# Mortality among children under five years admitted for routine care of severe acute malnutrition: a prospective cohort study from Kampala, Uganda

**DOI:** 10.1186/s12887-020-02094-w

**Published:** 2020-04-24

**Authors:** Damalie Nalwanga, Victor Musiime, Samuel Kizito, John Baptist Kiggundu, Anthony Batte, Philippa Musoke, James K. Tumwine

**Affiliations:** 1 Department of Paediatrics and Child Health, School of Medicine, College of Health Sciences, Makerere University, P. O. Box 7072, Kampala, Uganda; 2 Research Department, Joint Clinical Research Centre, P. O. Box 10005, Kampala, Uganda; 3 Clinical Epidemiology Unit, Department of Internal Medicine, School of Medicine, College of Health Sciences, Makerere University, P. O Box 7072, Kampala, Uganda

**Keywords:** Severe acute malnutrition, Mortality, Children, Uganda

## Abstract

**Background:**

Mortality among children under 5 years of age admitted to malnutrition units in sub-Saharan Africa remains high. The burden of HIV infection, a major risk factor for mortality among patients with severe acute malnutrition (SAM), has reduced due to concerted prevention and treatment strategies. None the less, anecdotal reports from the malnutrition unit at Uganda’s National Referral Hospital (NRH) indicate that there is high mortality among patients with severe acute malnutrition (SAM) in routine care. Uganda has recently adopted the revised World Health Organization (WHO) treatment guidelines for SAM to improve outcomes. The mortality among children with SAM in routine care has not been recently elucidated. We report the magnitude and factors associated with mortality among children under 5 years of age admitted to the NRH for routine care of SAM.

**Methods:**

This was a cohort study of all severely malnourished children admitted to the NRH between June and October 2017. The primary outcome was two-week mortality. Mortality was calculated using simple proportions and Cox regression analysis was used to determine factors associated with time to mortality. Data was entered into Epidata and analysed using Stata v14.

**Results:**

Two-hundred-sixty (98.5%) children: 59.6% male; mean age 14.4 (SD 9.4) months, completed two weeks of follow-up. Of these, 25.2% (95% CI 19.9–30.4%) died. In-hospital mortality was 20.7% (95% CI15.9–25.6%). The prevalence of HIV infection was 12.2%. Factors associated with mortality included: positive HIV status (AHR 2.2, (95% CI; 1.2–4.2), *p* = 0.014), bacteraemia (AHR 9 (95% CI 3.4–23.0), *p* < 0.001, and low glomerular filtration rate (eGFR), AHR 3.2; (95% CI 1.7–6.3), *p* = 0.001).

**Conclusions:**

A 25% mortality among children with severe malnutrition remains unacceptably high despite significant reduction in HIV prevalence. Children with SAM who are HIV infected, have eGFR below 60 mL/min/1.73m^2^ or have bacteraemia, are more likely to die. Further studies to explore the relationship between eGFR and mortality among children with SAM are needed. Studies to establish efficacious antibiotics are urgently required to inform treatment guidelines for children with SAM.

## Background

Severe acute malnutrition (SAM) adversely affects the lives of children under 5 years of age in low-income countries. Increasingly malnutrition is recognized as one of the leading contributors to the burden of disease in these countries [1, 2]. Over 20 million children under the age of 5 years are affected by SAM with an estimated annual mortality of 1–2 million in sub-Saharan Africa alone [3]. In Uganda, 4% of children under the age of 5 years are wasted, and 1 % of these are severely wasted [4]. In 2006, 7 % of children under 5 years of age admitted at the Acute Care Unit (ACU), Mulago hospital (the national referral hospital in Uganda) had SAM. These children experienced a 24% mortality, with the majority of deaths occurring in the first week of admission [5].

Recent publications from the MNU at Mulago Hospital indicate that mortality among children admitted for SAM ranges from 9.8 to 25% [5]. These numbers are dependent on whether children were enrolled in a clinical trial, were relatively stable or were under routine care [6, 7].

Mortality among SAM patients under routine care in Mulago Hospital was last assessed objectively in 2006 [5]. The WHO guidelines for inpatient management of children with SAM were revised in 2013 and have contributed to significant reduction in mortality in treatment centres across sub-Saharan Africa [8]. Despite adopting these guidelines, anecdotal reports suggest that Mulago Hospital continues to register high mortality among these children. HIV infection is associated with increased mortality among children with SAM [9, 10]. In Uganda, children with SAM are routinely screened for HIV, and access to lifesaving antiretroviral therapy (ART) is now widely available. Although ART improves outcomes among children with SAM [9], mortality among children in routine care at Mulago Hospital in the ART era has not been evaluated. Other factors known to be associated with mortality in children with SAM include excess or unnecessary blood transfusion, and co-morbidities including diarrhoea, pneumonia, hypoglycaemia and bacteraemia [5, 7, 9, 11, 12]. The mortality of children with SAM admitted to MNU for routine care has not been clearly documented, and the factors associated with these mortalities have not been recently evaluated. Hence we report mortality and its associated factors in children under 5 years of age admitted to Mulago hospital for routine care of SAM.

## Methods

### Study setting

The study was conducted at the emergency ward/ acute care unit (ACU) and MNU at Mulago National Referral Hospital. The hospital receives patients from urban and peri-urban areas of Kampala and neighbouring districts as well as those further away referred for further management. The SAM patients are often critically ill, requiring emergency resuscitation at the ACU and 24 h of observation before transfer to the MNU for treatment and nutritional rehabilitation. Patients in the study received routine standard care for SAM as per the WHO guidelines at ACU and MNU by the clinical team. This includes treatment and prevention of hypoglycemia, hypothermia, dehydration, electrolyte imbalance, treatment of infection with antibiotics (1st line Ampicillin and Gentamycin and second line ceftriaxone unless otherwise indicated at clinicians’ discretion), correction of micronutrients with multivitamins and minerals, feeding (F75 and transitioned to ready to use therapeutic feed (RUTF) when they were clinically stable and passed an appetite test) and sensory stimulation. Children less than 6 months received F75 in addition to breast milk during admission and were discharged on breast milk and formula feeds where feasible. A diagnosis of dehydration was made by clinicians based on local guidelines [13]. All clinical and laboratory procedures were done within a 1 km radius of the Mulago Hospital complex.

### Study design

This was a prospective cohort study of 270 children under 5 years of age with SAM admitted to Mulago National Referral Hospital in Kampala, Uganda between June and October 2017. All children less than 5 years of age with very low weight for height (below − 3 Z scores of the median WHO growth standards [14]), visible severe wasting (mid upper arm circumference (MUAC) < 11.5 cm) or nutritional oedema, and whose caregivers gave written informed consent were enrolled consecutively.

### Study procedure

The study team included two pre-trained research assistants (a nurse and a medical officer) and the first author. Children admitted to the ACU with SAM within the previous 24 h were assessed for eligibility. They were assessed for danger signs such as altered level of consciousness, convulsions, dehydration, hypothermia or hyperthermia. Children with danger signs were resuscitated appropriately. The weight, height/length, weight for height/length, MUAC and presence of oedema were then re-checked to ensure the child met the criteria for the diagnosis of severe malnutrition. Informed consent was obtained from the parent or primary caregiver of the child who met the study criteria. A structured questionnaire was administered to the caregivers to obtain the child’s history.

Blood samples from all the eligible children were obtained at enrolment for complete blood count (CBC), serum electrolytes, urea, creatinine, liver enzyme tests, blood culture, malaria blood slide, random blood sugar (RBS) and HIV serology or DNA PCR (for infants < 18 months of age whose mothers tested positive on HIV serology). The blood samples were delivered to the laboratory within 2 h of collection. Estimated glomerular filtration rate (eGFR) was calculated using Schwartz formula [15]. The results of laboratory investigations were captured in the questionnaire and a copy was provided to the clinical team caring for the patients. Each participant had a chest X-ray done within 3 days of admission, which was read by two radiologists. The two radiologists discussed, agreed, and produced one report. If they did not agree, a third radiologist acted as a tie breaker. The CBC, serum electrolytes and malaria slide were done in the Mulago Hospital central laboratory, which is a WHO 3-star laboratory. Blood cultures were done using the BACTEC culture system, in the Makerere University College of Health Sciences Medical Microbiology laboratory, which is certified by the College of American Pathologists (CAP). The rapid HIV serology tests were done in the MNU ward side laboratory. Blood samples for HIV DNA-PCR tests were sent to Baylor Uganda, Centre of Excellence (COE)/Mulago Paediatric Infectious Diseases Clinic (PIDC).

The participants were followed-up for 2 weeks. The two-week period was chosen because previous studies among children with SAM have demonstrated that 75% of deaths occur in the first week and up to 90% by end of the second week [5]. Follow-up data was captured in the participant questionnaire. Participants were censored if they survived until day 14 of follow up or earlier if they were lost to follow up.

### Data management

The questionnaires were completed by study staff and reviewed weekly for accuracy and completeness prior to data entry. Data was entered into an electronic database using Epidata version 3.1 software package with built-in quality control checks. The data was double-entered and validated by two entrants. The final data was backed up and exported to Stata version 14.1 (STATA CORP, TEXAS USA) for analysis. Continuous variables were summarized using means and standard deviations for normally distributed data. Categorical variables were summarized using frequencies, and percentages. Mortality proportion and rate were determined. Time to mortality was also determined. We performed Cox regression analysis to determine factors associated with time-to-mortality. Bivariate analysis was performed for each of the independent variables to determine whether they were independently associated with mortality. The strength of the association was assessed using hazard ratios (HR) and 95% confidence intervals. Multivariate analysis was performed to determine whether the independent variables were jointly associated with the outcome. Variables with a *p*-value of ≤0.2 at bivariate analysis were considered for the multivariate model. The variables were entered into a stepwise model. Interaction between the variables which remained in the model was assessed using the Chunk test. This was followed by assessing for confounding using a difference of ≥10% between the crude and adjusted measure of effect (HR) for the variables that would have gone out at each step. Significance was set at *p* value of 0.05 or less. A Kaplan Meier curve was drawn and the differences between groups were determined using the Log rank test.

## Results

### Participant clinical and demographic characteristics

**Fig. 1:**
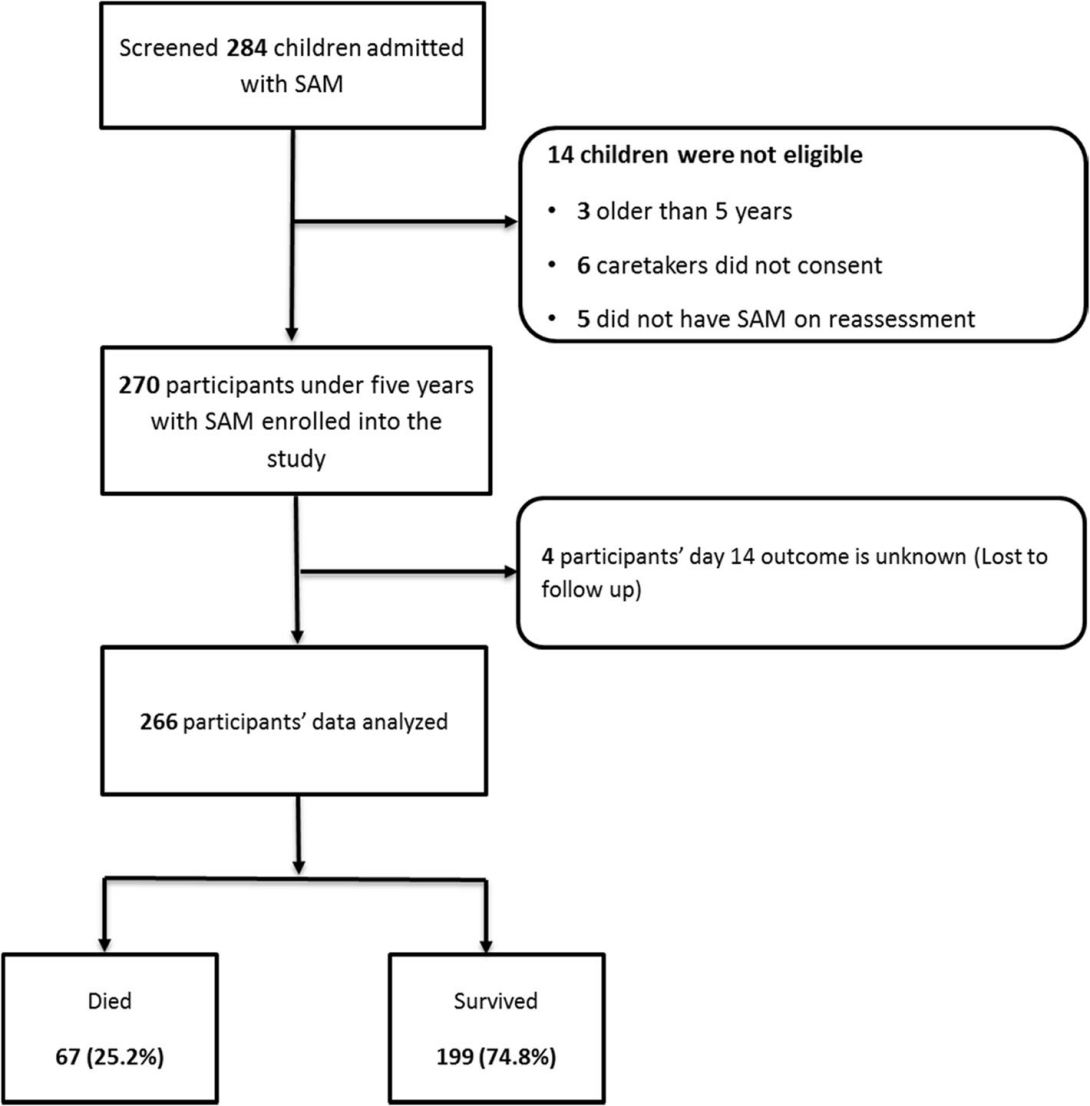
Study profile for recruitment of 270 children admitted for routine care of SAM

A total of 270 children below 5 years of age with SAM were recruited, as shown in Fig. 1. The mean age was 14.4 months (SD9.4) and 161/270 (59.6%) were male. Thirty-three (12.2%) children were under 6 months of age and 229 (85.9%) were under 24 months. Most of the 270 caregivers were the participants’ mothers (78.5%) with a mean age of 27.4 years (SD8.3) and 11.3% below 19 years of age. Many of them (36.6%) were housewives and the majority (57.8%) had attained primary level education.

The majority (90%) of the participants had a recent history of weight loss, cough (191/270; 70.7%), diarrhoea (151/270; 55.9%) and fever (142/270; 52.6%) prior to hospitalization. Thirty-four (12.6%) had chronic medical illnesses, including congenital heart diseases and cerebral palsy. Forty-one (15.2%) of participants had documented evidence of having received medication for their current presentation prior to admission. Visible wasting, pallor, dermatosis and dehydration were observed in many of the participants. One-hundred and twenty-three (45.6%) of the participants had oedematous SAM. Nine (3.4%) participants had hypothermia. Seven participants were admitted in shock (Table 1). Overall, 33 (12.2%) children were HIV-infected; 16 (48.5%) of these were identified prior to enrolment while 17 were diagnosed during the study. Of the HIV-infected children identified prior to enrolment, 10 (62.5%) were receiving anti-retroviral therapy (ART). Mean duration of ART was 8 days (SD 9.7)**.** The total number of HIV-exposed infants (infected and uninfected) was 40/189 (21.2%); of these, 22 (55%) were HIV-exposed but uninfected (HEU). The prevalence of HIV in the oedematous children was 11/123 (8.9%) compared to22/147 (15%) in the non-oedematous group, *p* = 0.132.

**Table 1:** History and clinical examination findings of children with severe acute malnutrition admitted to Mulago hospital at enrolment

Variable	n/N	Percentage
Weight loss	243	90
Cough	191	70.7
Diarrhea	151	55.9
Fever	142	52.6
Appetite loss	133	49.3
Vomiting	127	47
Skin rash	64	23.7
Convulsions	26	9.6
Loss of consciousness	5	1.9
Difficulty in breathing	47	17.4
Abdominal pain	57	21.1
Medical history		
Chronic illness excluding HIV^5^	34	12.6
Received treatment prior to admission	41	15.2
Temperature (°C): *N* = 263		
Normal (35–37.4)	177	67.3
Hypothermia (< 35 °C)	9	3.4
Hyperthermia (≥37.5 °C)	77	29.3
MUAC^a^ (cm): *N* = 237		
Less than 11.5	138	58.2
11.5 and above	99	41.8
Weight for Height/ Length (Z score)		
≤ −3	210	77.8
> −3	60	22.2
Signs of Vitamin A deficiency	5	1.9
Grade of edema^b^		
Grade I	22	17.9
Grade II	45	36.6
Grade III	56	45.5
Shock	7	2.6
Dehydration	98	36.3
Visible wasting	212	78.5
Dermatosis	119	44.1
Thrush	61	22.6
Pallor (palmar and/or conjunctiva)	143	53
Jaundice	7	2.6
Signs of Respiratory Distress	48	17.8
Abnormal breath sounds^c^	44	16.4
Abnormal heart sounds^d^	7	2.6
Abnormal motor findings	21	7.8
Abdominal Distension	57	21.1

Chronic illness include congenital heart defects, cerebral palsy, epilepsy, and hernia, *N* = 270, unless otherwise stated due to missing data, ^a^*MUAC* Mid Upper Arm Circumference, ^b^*N* = 123 (nuber of children with oedema)^c^ Abnormal breath sounds: rhonchi, crepitations; ^d^ Abnormal heart sounds: murmurs

Ten (3.7%) of the participants had hypoglycaemia in the first 24 h of admission. Mean total white cell count was 14.1 X 10^3^ ± 7.4 cells/μL, with mean differential neutrophil and lymphocyte count of 40.5 and 49.1% respectively. Thirty-six percent of the participants had severe anaemia (haemoglobin < 7 mg/dL) while 9 (3.3%) had anaemia requiring blood transfusion (haemoglobin < 4 mg/dL). Eight (3%) of the 270 blood cultures had bacterial growth. The isolated organisms included *E.coli* (2), *Candida species* (1), *Salmonella spp* (1), *Streptococcus pneumoniae* (2), *Citrobacter freundii* (1), and *Rhodococcus Spp* (1). Five of the 7 bacterial isolates were resistant to ampicillin, 1 to gentamicin and 4 to ceftriaxone. None was resistant to both ampicillin and gentamycin. Of the 183 patients that had chest X-rays, 76 (41.5%) had abnormal results (Table 2).

**Table 2:** Assessment findings of children with severe acute malnutrition admitted to Mulago hospital

Variable	n/N	Percentage
Glucose levels (mmol/l): ***N*** = 268		
< 3	10	3.7
3- < 8.3	215	80.2
≥ 8.3	43	16.1
Hemoglobin levels (g/dl): ***N*** = 266		
< 7	36	13.4
7.0–9.9	132	49.5
10–10.9	46	17.2
> 11	52	19.9
Total WBC: ***N*** = 266		
Abnormal	144	54.1
Neutrophils: ***N*** = 266		
Abnormal	135	50.8
Albumin (g/l): ***N*** = 266		
< 3.5	80	30.1
Total protein (g/l): ***N*** = 250		
< 63	207	82.8
ALT(U/L): ***N*** = 266		
Elevated	99	37.2
ALP (U/L): ***N*** = 259		
Elevated	222	85.7
GFR^b c^ (mL/min/1.73m^2^): ***N*** = 261		
< 30	16	6.1
30- < 60	46	17.6
60- < 90	55	21.1
≥ 90	144	55.2
Sodium (mmol/dl): ***N*** = 266		
< 135	77	29
Potassium (mmol/dl): ***N*** = 266		
< 3.5	66	24.9
Calcium (mmol/dl): ***N*** = 265		
< 2.2	226	85.3
Phosphate (mmol/dl): ***N*** = 260		
< 1.28	108	42
Blood smear for Malaria		
Positive	15	5.6
Blood culture		
Positive blood culture	8	3
Chest x-ray: (*N* = 183)		
Abnormal^a^	76	41.5
Infiltrates	61	80.3
Consolidation	15	19.7
Hyperinflation	10	13.2
HIV status		
Total HIV positive	33	12.2
HIV exposed: *N* = 189	40	21.2
HIV exposed –infected	18	45
HIV exposed –Uninfected	22	55

*N* = 270, unless otherwise stated due to missing data, ^a^Some participants had more than one abnormality, ^b^GFR (Using Schwatz formula) = (K^c^height)/cr (mg/dl), (http://nephron.com/bedside_peds_nic.cgi)

### Mortality among the participants

The overall mortality of the children in the study was 67/266 (25.2%; 95% CI 19.9–30.4%). The overall mortality rate was 2.4 deaths per 100 person days of follow-up. In-hospital mortality was 56/270 (20.7%) during the 14 days of follow-up (Table 3). Eight (11.9%) participants died in the ACU, 44 (65.7%) died in MNU, 4 (6%) died on general paediatric wards, and 11 (16.4%) died at home following discharge against medical advice. No participants died during transfer to MNU. Twenty (29.85%) of the mortalities occurred within 48 h of admission, 27 (40.30%) within 3–7 days of admission and 20 (29.85%) between 8 and 14 days of admission. Thirty-four (60.7%) of the participants died during day shifts while 22 (39.2%) died at night i.e. 8 am and 8 pm. The most common diagnoses around the time of death were shock (20.9%), acute watery diarrhoea (13.4%), aspiration pneumonia or bronchopneumonia (11.9%), and pulmonary TB (9%). Of the 147 HIV-unexposed infants, 35 (23.8%) died, compared to 8 of the 22 (36.4%) in HEU group, *p* = 0.21.

**Table 3:** Mortality among children with severe acute malnutrition admitted to Mulago hospital

Variable	Dead n/N^a^	% (95% CI)	P value
Age (months)			
< 6	8/32	25 (0.1–0.4)	0.990
6–23	50/197	25.4 (0.2–0.3)	
≥ 24	9/37	24.3 (0.1–0.4)	
HIV status			
Positive	15/32	46.9 (28.6–65.2)	**0.003**
Negative	42/234	22.2 (16.9–27.6)	
HIV status awareness at admission			
Positive and aware at admission	10/15	66.7 (6.3–60.4)	**0.035**
Positive and not aware	5/17	29.4 (5.3–53.6)	
ART status at admission			
On ART	7/10	70 (4.6–64.6)	0.077
Not on ART	8/22	36.4 (14.5–58.2)	
HIV exposure			
HEI and HIV negative			
HEI	9/17	47.1 (23.5–72)	**0.004**
HIV negative	52/234	22.2 (17.3–28)	
HEU^c^ and HIV negative			
HEU	8/22	36.4 (40.5–81.8)	0.094
HIV negative	44/212	20.8 (15.7–26.8)	
Edematous			
Edematous	23/122	18.9 (11.8–25.9)	**0.028**
None	44/144	30.6 (22.9–38.2)	
Diarrhea			
Yes	24/116	28.7 (64–78.7)	0.137
No	43/150	20.7 (13.2–28.1)	
Hemoglobin levels (g/dl)			
< 7	12/36	33.3 (17.2–49.5)	0.25
≥ 7	55/226	24.3 (18.7–30)	
Hemoglobin levels (g/dl)			
< 4	6/9	66.7 (28.2–71.7)	**0.004**
≥ 4	61/253	24 (18.8–29.4)	

^a^N=Number of participants in each category whose outcome was known, ^b^ One of the HIV positive patients was lost to follow up, ^c^ One of the HEU children was lost to follow up

**Fig. 2:**
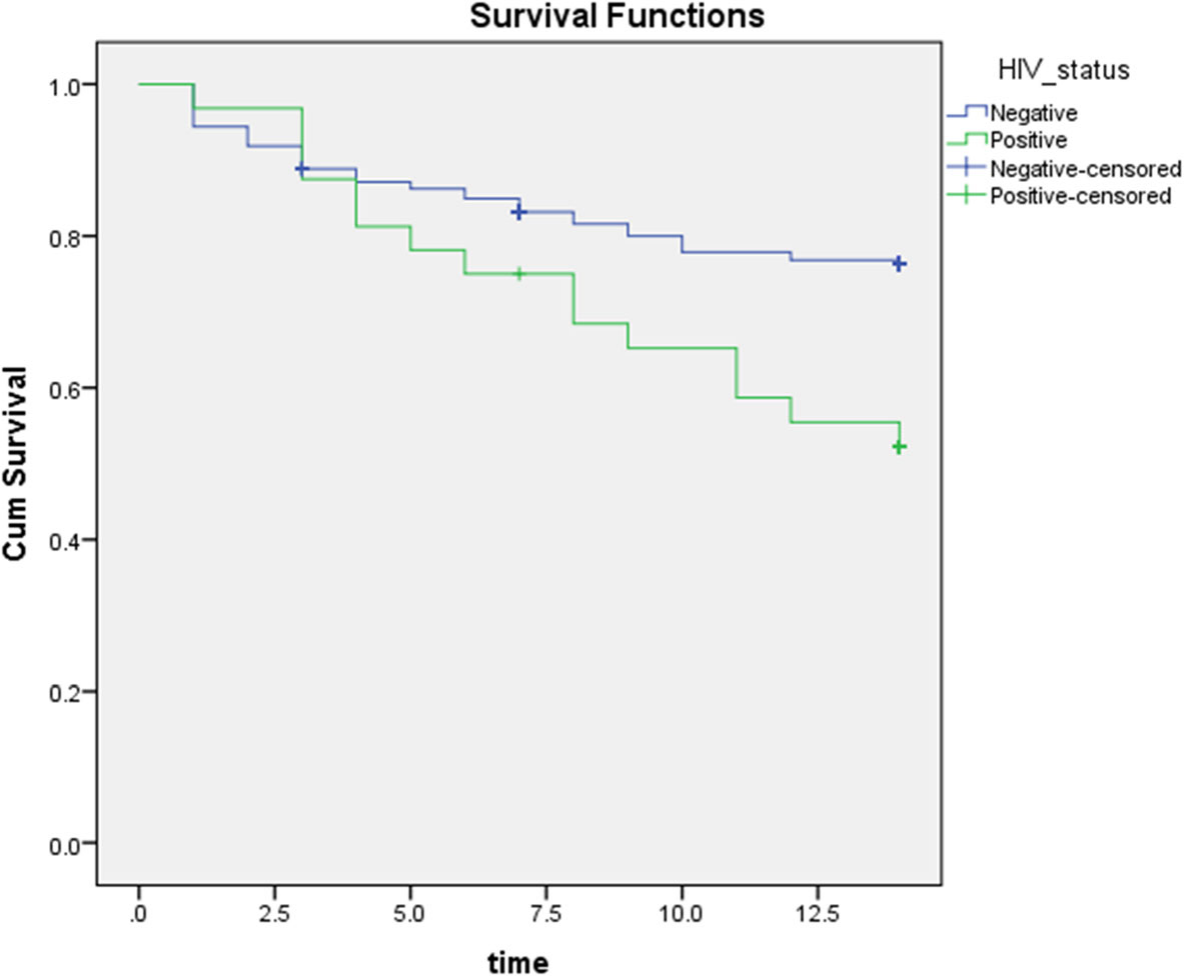
Kaplan Meier survival curve for children admitted with SAM and HIV positive versus HIV negative

The participants’ survival time was 11.7 days (11.3–12.4) on average. The Kaplan Meier survival curve for children with and without HIV is shown in Fig. 2. The survival time was significantly lower among the HIV-positive participants (10.6, CI; 8.9–12.2 days) compared to their HIV-negative counterparts (11.9, CI; 11.3–12.4 days) with a *p* value of 0.008.

Significant factors associated with survival time at multivariate analysis included; positive HIV status (AHR 2.2, 95% CI; 1.2–4.2, *p* = 0.014), positive blood culture (AHR 9; 95% CI 3.4–23, p = < 0.001), and low estimated glomerular filtration rate (eGFR), (AHR 3.2; 95% CI 1.7–6.3, *p* = 0.001), as summarised in Table 4.

**Table 4:** Bivariate and multivariate analysis for factors associated with time to mortality (Cox regression analysis) among children with severe acute malnutrition admitted to Mulago hospital

Variable	Dead n/N (%)	Bivariate analysis	*p* value	Multivariate analysis	*p* value
		UHR (95% CI)		AHR (95% CI)	
Sex					
Male	38/160 (23.8)	1			
Female	29/106 (27.4)	1.2 (0.7–1.9)	0.505		
Diarrhea					
No	24/116 (20.7)	1		1	
Yes	43/150 (28.7)	1.5 (0.9–2.4)	0.142	1.1 (0.7–1.9)	0.684
Fever					
No	28/127 (22.1)	1			
Yes	39/139 (28.1)	1.3 (0.8–2.1)	0.339		
Temperature (°C)					
Normal	38/175 (21.7)	1			
Hypothermia	2/9 (22.2)	1.02 (0.2–4.2)	0.977		
Hyperthermia	26/75 (34.7)	1.6 (1–2.7)	0.059		
Dehydration					
No	33/168 (19.6)	1			
Yes	34/98 (34.7)	2 (1.2–3.2)	0.005		
Oral thrush					
No	43/206 (20.9)	1			
Yes	24/60 (40)	2.1 (1.2–3.4)	0.005		
Edema					
No	44/144 (30.6)	1		1	
Yes	23/122 (18.9)	0.6 (0.4–1)	0.050	1 (0.6–1.8)	0.951
Visible Wasting					
No	10/57 (17.5)	1		1	
Yes	57/209 (27.3)	1.6 (0.8–3.1)	0.168	1.3 (0.6–2.6)	0.493
Signs of vitamin A deficiency					
No	65/262 (24.8)	1		1	
Yes	2/4 (50)	3.2 (0.8–13.3)	0.102	1.5 (0.3–7.5)	0.601
Diagnosis of pneumonia					
No	33/162 (20.4)	1		1	
Yes	34/104 (32.7)	1.7 (1.1–2.8)	0.027	1.5 (0.9–2.5)	0.153
HIV status					
Negative	52/234 (22.2)	1		1	
Positive	15/32 (46.9)	2.1 (1.2–3.8)	0.01	2.2 (1.2–4.2)	0.014
Blood culture					
No growth	61/258 (23.6)	1		1	
Growth	6/8 (75)	6.3 (2.7–14.9)	0.000	9 (3.4–23.4)	< 0.001
Glucose levels					
< 3	4/10 (40)	2.4 (0.9–6.7)	0.097	2.1 (0.7–6.3)	0.163
3- < 8.3	41/211 (19.4)	2.9 (1.7–4.8)	0.000	1	
≥ 8.3	22/43 (51.2)			1.5 (0.9–2.7)	0.154
GFR					
≥ 90	23/144 (16)	1		1	
60- < 90	12/55 (21.8)	1.4 (0.7–2.8)	0.340	1.6 (0.8–3.3)	0.188
30- < 60	19/46 (41.3)	3.1 (1.7–5.7)	0.000	3.2 (1.7–6.3)	0.001
< 30	12/16 (75)	6.5 (3.2–13.1)	0.000	5.2 (2.3–11.4)	< 0.001
Potassium (mmol/dl)					
≥ 3.5	43/195 (22.1)	1		1	
< 3.5	23/(34.9)	1.7 (1–2.8)	0.046	1.3 (0.8–2.3)	0.336
Hemoglobin level (g/dl)					
≥ 7	55/226 (24.3)	1			
< 7	12/36 (33.3)	1.3 (0.7–2.5)	0.351		

## Discussion

This study investigated mortality and associated factors among 270 children under five years of age admitted to Mulago Hospital for care of severe acute malnutrition (SAM). The main findings were an overall mortality of 25% which is higher among children with HIV infection, positive blood cultures and low estimated GFR. Age, diarrhoea, hypoglycaemia, hypothermia, electrolyte imbalance, pneumonia and oral thrush were not associated with mortality in this cohort of children with SAM.

This mortality is similar to the 24% recorded by Bachou et al. over ten years earlier among patients receiving the standard of care at Mulago hospital [5]. However, the mortality recorded in this study is much higher than that reported by more recent studies conducted in selected populations: 14% in 2017 and 9.8% in 2018 by Rytter et al. and Nabukeera et al. respectively [6, 7]. Our observational study included all severely malnourished children receiving routine care. Nabukeera et al.’s study was a nested randomised control study (RCT) including a selected population with frequent patient monitoring and access to more resources. Rytter et al.’s study was an observational study but excluded critically ill children.

The mortality in our study is higher than that recorded in retrospective studies from South Sudan (9.3%) and the Democratic Republic of Congo (DRC)(7.5%) [16, 17]. A prospective study in Malawi reported a comparable mortality of 18% [18]. The higher mortality in our population could be explained by the fact that our study was done in a tertiary referral hospital. In addition, we followed up patients discharged against medical advice. The higher HIV prevalence in Uganda compared to South Sudan and DRC could also explain the difference. Patients in tertiary hospitals are often the most severely ill patients following referral from smaller centres. Eleven of the study participants died at home following discharge against medical advice; which likely contributed to the high mortality. This finding is not surprising considering that a number of children in Uganda and Malawi are documented to die at home following official medical discharge [10, 19].

Mortality among children with SAM in Mulago is still significantly higher than the set targets of 5 and 10% by the WHO and Sphere standards, respectively, yet the findings in RCTs suggest the mortality can significantly be improved. Most of the deaths, 42 (67%) in this study occurred in the first week of admission, which is consistent with other observational studies in the same population and sub-Saharan Africa [5, 6, 20, 21]. Closer patient monitoring in the first week of admission could bring us closer to these targets.

HIV-positive children had a significantly shorter survival time than HIV-negative children. These findings are similar to the systematic review done by Fergusson et al., and observational studies in Uganda and Malawi [6, 9, 10, 22–24]. A significant number of HIV-positive children in this study were diagnosed prior to admission. However, our findings are different from those of Nabukeera et al. where the mortality was lower among HIV-positive children on ART [6].

Children in the ART group in this study tended to have a higher mortality when compared to the ART naïve group. This finding could be attributed to the Immune Reconstitution Inflammatory syndrome (IRIS) which occurs in the early days of ART initiation since the mean duration on ART in this group was 8 (SD 9.7) days. Alternatively, these children may have been more severely ill prior to admission, resulting in investigation for HIV and initiation of ART, compared to the ART naïve children who had not yet been identified. In this study, there was no difference in mortality among the HIV exposed infected (HEI) and HIV exposed uninfected (HEU) infants, possibly due to the small numbers. Similarly there was no difference between the HIV unexposed uninfected (HUU) and the HEU. On the other hand, a study in Uganda comparing morbidity between HEU and HUU children showed a higher risk of SAM among the HEU group, partially attributed to early breastfeeding cessation [25]. Despite the reduction in prevalence of HIV among children with SAM from 40 to 50% in 2006–2009 [5, 11], to less than 15% in 2016–2018 [6, 7] and 12.2% in this study, mortality from SAM has remains high and HIV has remained an important contributor to these deaths. Prevention of HIV, early initiation of ART, nutritional support for HIV-positive children, and close monitoring of HIV-positive children on ART to prevent malnutrition and its complications could help reduce severe malnutrition, which is associated with poor outcomes.

A positive blood culture was associated with a significantly reduced survival time among these children. Children with SAM are expected to have severe infections due to the immune suppression caused by malnutrition [26]. Although the prevalence of bacteraemia in this study was lower than that reported in other studies [11, 27–29], it still significantly influenced mortality among children with SAM. Only 15% of the participants in this study reported receiving treatment prior to admission. Only those with written evidence of previous treatment were included in this category. We, however, suspect that more children received antibiotics prior to admission as Mulago hospital is a tertiary centre where difficult cases from other centres are referred after failing to respond to treatment. Such treatment regularly includes antibiotics, but this is often not documented. Furthermore, use of non-prescription antibiotics is a common practice in low-income countries like Uganda and contributes to antibiotic resistance [30]. This might explain the low prevalence of bacteraemia and might also suggest that the patients who had bacteraemia most likely had overwhelming sepsis or were colonised by bacteria strains that were resistant to the antibiotics they had received. Such children are therefore more likely to die compared to those who might have been infected with susceptible bacteria. Contrary to other studies in which the most frequently isolated organism was *Staphylococcus aureus*, the isolated organisms in this study were, *Escherichia coli, Streptococcus pneumoniae*, *Candida species, Salmonella species, Citrobacter freundii,* and *Rhodococcus species* [27, 29]*.* Resistance to WHO-recommended ampicillin was very high (71.4%), a finding that is consistent with other studies of SAM children in Africa [11, 27–29, 31]. Resistance to ceftriaxone was also high (57.1%). This calls for further evaluation of the optimal antibiotics for use among children with SAM.

The survival time of children with a low eGFR below 60 mL/min/1.73m^2^ was 3.2 times less than that of those with eGFR more than 60 mL/min/1.73m^2^. There is a paucity of data on the relationship between poor kidney function and survival among children with malnutrition. However, studies have demonstrated that kidney injury is a risk factor for mortality in children who are critically ill and those with malaria [32, 33]. In our study, even after controlling for factors that indicate compromised perfusion (dehydration, shock, diarrhoea), reduced eGFR remained an independent predictor of reduced survival time. A study of 32 malnourished Jamaican children reported reduced eGFR, which normalised as they recovered from malnutrition. Only a single serum creatinine test at admission was done in our e study, therefore we cannot ascertain whether children developed acute kidney injury or had pre-existing chronic kidney disease. However, acute kidney injury in these children may result from hypovolemia following diarrhoea and vomiting, sepsis, renal toxic medications such as gentamicin, or non-steroidal anti-inflammatory agents.. The relationship between malnutrition and reduced eGFR and its impact on mortality among children with SAM needs to be explored further as it may have significant implications on the management of these children.

Other factors associated with mortality in previous studies including diarrhoea, hypoglycaemia, hypothermia, electrolyte derangements, pneumonia and oral thrush among others were not significant in this study. This may be attributed to the difference in severity of these factors among the participants in this study compared to other studies. Some factors including hypothermia and hypoglycaemia occurred infrequently in this study, (9/263, 3.4%) and (10/269, 3.7%) respectively and may not have reached significance as a result. The correlation between HIV and diarrhoea resulting in interaction may explain why diarrhoea was not significant in this study.

The strengths of the study include: the prospective cohort study design, which is ideal for establishing temporal relationship between mortality and associated factors. The study investigated factors associated with time to mortality which not only guides clinicians on which patients need close follow-up but also helps identify those whose interventions are time-sensitive. The sample size was sufficient to answer the study questions, and participants who left the hospital against medical advice were followed up, thus significantly reducing the rate of loss-to-follow-up. The main limitations include: the results cannot be generalized to all centres in Uganda since the study participants were recruited from a tertiary referral site that may include more severely ill children than those from other facilities. On the other hand, they provide insights into management of children with SAM in hospitals with a similar set-up in other countries within sub-Saharan Africa. All laboratory tests were done once on admission and chest x-rays were not done for a number of the participants due to lack of consent, and frequent dysfunction of the x-ray machine. The study assessed the association between admission characteristics and survival time, however these could have changed during follow-up. We cannot comment on the causes of death for these children as autopsies were not done.

## Conclusions

One in four children under five years of age admitted to Mulago hospital with SAM died, which is unacceptably high. Factors associated with reduced time-to-mortality of children under five years admitted with SAM were HIV infection, bacteraemia, and eGFR below 60 mL/min/1.73m^2^. These children should be more closely monitored. Studies to explore the causes of reduced eGFR as well as the relationship between eGFR and mortality among children with SAM are required. Large studies to identify the causative organisms for bacteraemia and sensitivity patterns among children with SAM are also recommended to inform antibiotic treatment guidelines for children with SAM.

## Data Availability

The datasets used and/or analysed during the current study are available from the corresponding author on reasonable request.

## Abbreviations

ACU: Acute Care Unit
ART: Anti-Retroviral Therapy
CBC: Complete blood count
eGFR: Estimated Glomerular Filtration Rate
HIV: Human Immunodeficiency Virus
HEU: HIV exposed uninfected
HEI: HIV exposed infected
IRIS: Immune Reconstitution Inflammatory Syndrome
MNU: Mwanamugimu Nutrition Unit
MUAC: Mid-upper arm circumference
NGT: Nasogastric tube
PI: Principal Investigator
PIDC: Paediatric Infectious Disease Centre
PMTCT: Prevention of Mother to Child Transmission
RA: Research Assistant
SAM: Severe Acute Malnutrition
SD: Standard Deviation
TB: Tuberculosis
UNICEF: United Nations International Children’s Emergency Fund
WFH: Weight for height
WHO: World Health Organisation
